# Potential Applications for Growth Hormone Secretagogues Treatment of Amyotrophic Lateral Sclerosis

**DOI:** 10.2174/1570159X20666220915103613

**Published:** 2023-09-25

**Authors:** Ramona Meanti, Elena Bresciani, Laura Rizzi, Silvia Coco, Vanessa Zambelli, Anna Dimitroulas, Laura Molteni, Robert J. Omeljaniuk, Vittorio Locatelli, Antonio Torsello

**Affiliations:** 1 School of Medicine and Surgery, University of Milano-Bicocca, Via Cadore 48, Monza, 20900, Italy;; 2 Faculty of Health and Medical Sciences, University of Surrey, Stag Hill, Guildford, GU2 7XH, United Kingdom;; 3 Department of Biology, Lakehead University, 955 Oliver Rd, Thunder Bay, Ontario, P7B 5E1, Canada

**Keywords:** Growth hormone secretagogues, ghrelin, amyotrophic lateral sclerosis, ALS, neuroinflammation, neurodegenerative diseases

## Abstract

Amyotrophic lateral sclerosis (ALS) arises from neuronal death due to complex interactions of genetic, molecular, and environmental factors. Currently, only two drugs, riluzole and edaravone, have been approved to slow the progression of this disease. However, ghrelin and other ligands of the GHS-R1a receptor have demonstrated interesting neuroprotective activities that could be exploited in this pathology. Ghrelin, a 28-amino acid hormone, primarily synthesized and secreted by oxyntic cells in the stomach wall, binds to the pituitary GHS-R1a and stimulates GH secretion; in addition, ghrelin is endowed with multiple extra endocrine bioactivities. Native ghrelin requires esterification with octanoic acid for binding to the GHS-R1a receptor; however, this esterified form is very labile and represents less than 10% of circulating ghrelin. A large number of synthetic compounds, the growth hormone secretagogues (GHS) encompassing short peptides, peptoids, and non-peptidic moieties, are capable of mimicking several biological activities of ghrelin, including stimulation of GH release, appetite, and elevation of blood IGF-I levels. GHS have demonstrated neuroprotective and anticonvulsant effects in experimental models of pathologies both *in vitro* and *in vivo*. To illustrate, some GHS, currently under evaluation by regulatory agencies for the treatment of human cachexia, have a good safety profile and are safe for human use. Collectively, evidence suggests that ghrelin and cognate GHS may constitute potential therapies for ALS.

## 1 INTRODUCTION

The pleiotropic effects of ghrelin and growth hormone secretagogues (GHS) on neurons and muscle cells suggest that these compounds could be developed for the treatment of amyotrophic lateral sclerosis (ALS).

Ghrelin is known as the “hunger hormone” and is endowed with several endocrine effects, such as the stimulation of growth hormone (GH) secretion [1]. However, research in the two last decades has shown its regulatory roles in many organs and systems and now it is clear that it has much broader functions. Even before the discovery of ghrelin, a large number of synthetic ligands of the GHS-R1a receptor, called growth hormone secretagogues (GHS), have been developed.

In the 40 years of GHS history, it has been demonstrated that ghrelin and synthetic GHS not only stimulate GH secretion but also improve cellular and systemic metabolism, exert neuroprotective, anticonvulsant and anti-inflammatory effects, and modulate cardiovascular function [2]. Moreover, they increase food intake and body weight and participate in the regulation of skeletal muscle mass in animals and humans by stimulating insulin-like growth factor 1 (IGF-1) production [1, 3, 4].

Focusing on ALS, given the elevated heterogeneity and complexity of its pathophysiological mechanisms, approved therapeutic strategies result in only minimal effects on the disease course and survival of patients. Moreover, ALS patients are accompanied by hypermetabolism, weight loss, altered appetite, muscle atrophy, mitochondrial dysfunction, and neurodegeneration, all mechanisms that could be mitigated by ghrelin and GHS.

Therefore, increasing evidence suggests that ghrelin and GHS could modify disease processes in ALS.

## 2 GHRELIN AND SYNTHETIC GHS

Ghrelin, an octanoylated 28-amino acid peptide produced by the oxyntic glands of the stomach, stimulates growth hormone (GH) secretion and appetite, resulting in body weight gain. The discovery of ghrelin was obtained by reverse pharmacology. In 1976 Bowers and colleagues synthesized different derivatives of met-enkephalin, which were devoid of opiate activity but stimulated GH release from the pituitary gland [2]. In 1982, Bowers and Momany [5] developed the growth hormone-releasing peptide-6 (GHRP-6), which selectively stimulated the release of GH. Afterwards, Howard and colleagues cloned the G-protein-coupled seven-transmembrane GH-secretagogue receptor (GHS-R1a) [6], which a few years later allowed for the identification of ghrelin as the endogenous ligand of GHS-R1a.

The growth hormone secretagogues (GHS) are a large family of synthetic compounds with a heterogeneous chemical structure, which includes peptidyl, peptidomimetic, and nonpeptidic moieties, originally developed for their capability to stimulate the physiologic pulsatile GH and IGF-1 secretion, both *in vitro* and *in vivo* [7]. This is achieved *via* direct growth hormone release hormone (GHRH) mimicry or through interactions with GHS-R1a receptors [8].

### 2.1 Ghrelin, GH and IGF-1 Levels in ALS Patients

Ghrelin is released primarily in two different forms, one acylated at the serine 3 position with an octanoyl group (C8:0) [9]. Acyl ghrelin is the active form of ghrelin, capable of binding the GHS-R1a to reach and cross the blood-brain barrier (BBB) and capable of increasing the secretion of GH and glucagon, reducing the secretion of insulin, and contributing to the maintenance of plasma glucose levels. Furthermore, acyl ghrelin stimulates appetite, regulates gastrointestinal motility, cardiac function, osteoblast proliferation, and myoblast outgrowth, and exerts a broad range of neuroprotective effects.

Unacylated ghrelin, also called desacyl ghrelin, is an isoform of ghrelin lacking esterification and is unable to bind the GHS-R1a receptor. Although desacyl ghrelin was generally considered an inactive product of ghrelin degradation, it has emerged to be an active peptide, having intriguingly physiological actions that can be similar or sometimes opposite to ghrelin [9].

Only very few studies have investigated the plasma levels of ghrelin in ALS. Acyl ghrelin was found to be significantly lower in patients with ALS than in control, which could justify the reduced total food intake, malnutrition, and reduced survival of ALS patients. In support of this, post-examination survival of ALS patients was shorter in the group with low plasma ghrelin levels than in the high-plasma ghrelin group, demonstrating that lower plasma ghrelin levels could be associated with poor prognosis [10].

In addition, dysfunctions in GH/IGF-I axis are present in most neurodegenerative diseases, among which is ALS, where patients show a dysregulated expression of GH and IGF-1 [11].

In particular, different studies have shown that GH/IGF-1 ratio in cerebrospinal fluid (CSF) and plasma are significantly lower in ALS patients in comparison with their control group [11-14]: these investigations demonstrate that ALS patients have a high prevalence of GH deficiency and at the same time increased levels of IGF-1. Therefore, although at the cellular level, IGF-1 generally acts as a pro-survival factor mediating the inhibition of the apoptosis pathway, under pathological conditions, IGF-1 seems to contribute to the pathogenesis of ALS. Although further studies are needed to better understand the underlying mechanism of GH/IGF-1 contribution to the onset and course of ALS, this evidence suggests that ghrelin and its synthetic analogues could be an interesting strategy to promote the restoration of the GH/IGF-1 axis.

## 3 AMYOTROPHIC LATERAL SCLEROSIS

Amyotrophic lateral sclerosis (ALS), or “Lou Gehrig’s disease”, is a motor neuron disease (MND) characterized by rapid deterioration of both upper and lower motor neurons in the brainstem, motor cortex, and spinal cord. Loss of motor neurons results in severe muscle atrophy, paralysis, and eventual respiratory insufficiency, typically leading to death within 3-5 years of disease onset [15].

ALS has been classically categorized into two different forms: the more common sporadic form (sALS, 90 to 95%) has no obvious genetically inherited component, whereas the familial-type (fALS, 5 to 10%) is more often associated with an autosomal dominant inheritance and, rarely, with a recessive and/or X-linked transmission [16]. However, this classification is now outdated because of the increasing knowledge of the complex genetic architecture of the disease [17].

The annual global incidence of ALS is estimated at 1.6 cases per 100,000 persons, with significant differential distribution according to location, ethnicity, and gender [18-20]. The incidence peaks between 60 and 75 years of age and typically occurs in Caucasian adults with a median age of onset of 64 years [18, 21]. ALS in adults younger than 30 represents only 5% of cases and is mainly associated with fALS [22]. Concerning gender effects, men present ALS 50% more often over their lifetime than women [23, 24].

### 3.1 Pathophysiological Mechanisms

#### 3.1.1 Molecular Mechanisms

ALS is recognized as a multifactorial disease involving genetic, molecular, and environmental factors; however, the specific mechanism underlying associated cell death is unknown.

Proposed hypotheses for the molecular pathogenesis of ALS include (i) glutamate excitotoxicity, (ii) structural and functional abnormalities of mitochondria, (iii) impaired axonal structure or transport defects, and (iv) free radical-mediated oxidative stress [21, 25, 26].

#### 3.1.2 Glutamate Excitotoxicity

The activity of glutamate receptors and transporters could be factors involved prominently in the contribution of glutamate excitotoxicity to ALS. Indeed, levels of excitatory amino acid transporters (EAATs) are significantly depressed in the motor cortex and in the spinal cord of ALS patients [27]. To illustrate, aberrant mRNA of isoform 2 of the astroglial glutamate transporter (EAAT2) was detected in ALS patients, thereby explaining depressed EAAT2 protein levels [28]. Low EAAT2 expression leads to an increase in extracellular concentration of glutamate, with consequent overstimulation of glutamate receptors and neuronal degeneration [21]. By contrast, experimental overexpression of EAAT2 (in a transgenic mutant model, EAAT2/G93A double transgenic mice) was associated with a delay in motor neuron degeneration and disease progression but was not associated with an increase in survival compared to SOD1(G93A) mice [29].

*In vitro* studies have also demonstrated that the administration of glutamate uptake blockers (*e.g*., threohydroxyaspartate, THA) mimics the chronic excitotoxicity mediated by defective glial and/or neuronal glutamate transport, as observed in ALS [30, 31].

The vulnerability of motor neurons to glutamate excitotoxicity, observed in ALS, may be due to higher membrane permeability for calcium (Ca^2+^), caused by unduly prolonged α-amino-3-hydroxyl-5-methyl-4-isoxazole-propionate (AMPA) receptor activation [32]. There is an indication that abnormal editing of the mRNA for subunit 2 of AMPA receptors (GluR2) may contribute to neuronal death in ALS patients [33]; selective AMPA receptor blockade was shown to reduce neuronal death in *in vitro* motor neuron cell culture [32].

#### 3.1.3 Mitochondrial Defects and Oxidative Stress

Appropriate mitochondrial operation and regulation are fundamental to cellular metabolism, Ca^2+^ homeostasis, control of apoptosis, and cell overall survival [34, 35].

Accumulating evidence indicates a correlation between mitochondrial morphological and biochemical abnormalities and ALS pathogenesis. This mitochondrial hypothesis was strengthened as a consequence of the identification of mutations in genes encoding proteins imported into the mitochondria, which results in mitochondrial DNA (mtDNA) defects [36, 37]. Furthermore, evidence from clinical studies also suggests that mitochondrial dysfunction may also be a factor contributing to ALS.

Histopathological observations of mitochondria from ALS patients and transgenic mouse models have revealed a correlation between mitochondrial dysfunction and morphological abnormalities, including (i) fragmented network, (ii) swelling, (iii) vacuolization, and (iv) augmented cristae in soma and proximal axons of skeletal muscle and spinal motor neurons [21, 38, 39].

This correlation between mitochondrial morphological anomalies and compromised mitochondrial operations in ALS patients appears widespread among CNS and extra-CNS tissues, including skeletal muscle and spinal motor neuron morphology [40, 41] and metabolism [42]. At the molecular level, chronic mitochondrial alterations induce apoptosis by cytochrome C release and the pro-apoptotic gain-of-function of the B-cell lymphoma 2 (BCL-2)-derived family of proteins, which directly contribute to neuromuscular degeneration and neuronal dysfunction [43].

Indeed, the elevation of free radicals and oxidative damage were found in post-mortem-tissues [44], cerebrospinal fluid (CSF), serum and urine of ALS patients [21]. In particular, ALS patients' fibroblasts *in vitro* have enhanced sensitivity to oxidative damage compared with those of non-ALS patient controls [45].

Nonetheless, oxidative stress is unlikely to be the only pathogenic determinant of ALS but is more likely to contribute to a vicious cycle that involves multiple and integrated molecular pathways.

#### 3.1.4 Impaired Axonal Transport

Motor neurons are highly polarized cells with extensive axons involved in action potential propagation, as well as bi-directional fast- and slow-axonal transport of organelles, RNAs, proteins and lipids. Defects in anterograde and retrograde [46] axonal transport are one of the early pathophysiological events in ALS. There is accumulating evidence obtained from post-mortem specimens from ALS patients of abnormal accumulations of neurofilaments, mitochondria, lysosomes, and axonal spheroids in both proximal and distal axons of motor neurons [46, 47]. Data from *in vitro* and *in vivo* studies using models of ALS reveal a reduction in slow and fast axonal transport and, consequently, significant spinal motor neuron loss, lower myelinated fibre densities, and muscle pathology [25, 48].

### 3.2 Genetics of ALS

Genes associated with ALS have been identified and mapped using genome-wide association studies (GWAS), next-generation sequencing (NGS) as well as classical genetic analytical techniques [49]. Indeed, the diagnosis of ALS includes not only the characterization of clinical features, as well as results from electrodiagnostic testing, but also analysis of the four most robust and commonly ALS-associated genes, to wit: (i) C9*orf*72, (ii) SOD1, (iii) TARDBP and, (iv) FUS. Other gene variants implicated in the etiology of ALS include: (i) ATXN2, (ii) UNC13A, (iii) ANG, (iv) SMN1 and, (v) SMN2 genes [50].

#### 3.2.1 C9orf72

The hexanucleotides expansion (GGGGCC) in a non-coding region of the C9*orf*72 gene in chromosome 9p21 is found to be the most frequent genetic cause of ALS [51].

ALS patients with C9*orf*72-related alterations manifest a late start of pathology and abbreviated survival duration. This motor neuron disease involves both upper and lower motor neuron dysfunction and frontotemporal lobar degeneration (FTLD). FTLD is accompanied by: (i) psychoses and hallucinations [52], (ii) changes in behaviour, (iii) some Parkinson-like symptoms and possibly, (iv) dementia [53].

#### 3.2.2 SOD1

The human superoxide dismutase 1 (SOD1) gene consisting of 5 exons and 4 introns, is localized in the 21q22 chromosome and encodes for superoxide dismutase Cu/Zn (a protein of 153 aa = 16kDa). An initial association of chromosome 21 mutations with ALS pathology was confirmed by Rosen in 1993 [54], and to date, more than 170 mutations have been identified in this gene [55].

Mutations of SOD1 result in several consequences, including (i) oxidative stress, (ii) mitochondrial alterations, (iii) modifications of gene expression with consequent abnormalities in protein interactions, (iv) formation of intracellular aggregates of misfolded SOD1 peptide, (v) neurofilamentary disorganization and cytoskeletal abnormalities, (vi) glutamate excitotoxicity, and (vii) activation of caspases including induction of apoptosis [56].

#### 3.2.3 TARDBP

TAR DNA binding protein (TARDBP), a gene of six exons located on chromosome 1p36, encodes the TDP-43 protein. TDP-43 is a ubiquitous RNA/DNA-binding protein of 414 aa involved in the regulation of gene expression, splicing and mRNA stability [57]. Alteration in the TDP-43 phosphorylation state causes protein aggregation and the development of inclusion bodies. TDP-43-related ALS has a clinical phenotype similar to that of SOD1 and presents spinal manifestations with lower motor neuron predominant pathology but without cognitive impairment [58].

#### 3.2.4 FUS/TLS

The FUS gene, located on chromosome 16p11, consists of 15 exons and encodes the DNA/RNA-binding protein FUS/TLS (fused in sarcoma/translocated in sarcoma). FUS/ TLS is a multifunctional protein of 526 aa, involved in gene expression and regulation, including transcription, RNA splicing and transport, DNA repair, and damage response [26].

Mutant FUS protein aggregates in large globular and elongated inclusion bodies in spinal cord motor neurons and dystrophic neurites' cytoplasm, which results in cellular toxicity and neurodegeneration [59].

The clinical presentation of FUS-related ALS is similar to that of classical ALS but is accompanied by a pronounced loss of strength in the extremities of the upper and lower limbs, but not in the bulbar region; in addition, onset occurs at young ages and is followed by an aggressive course, accompanied rarely by cognitive defects [60]. Heterozygous mutations in the FUS gene may cause hereditary essential tremor-4 (ETM4) [61], and in rare cases, cognitive impairment [62].

### 3.3 Muscle Degeneration in ALS

Skeletal muscles are the “end-organ” affected in ALS; indeed, muscle weakness, atrophy, and denervation (with consequent paralysis) are the hallmarks of ALS. It is commonly recognized that the selective loss of upper motor neurons in the motor cortex and lower motor neurons in the brainstem and spinal cord are the early events in ALS. However, beyond the role of motor neurons, accumulating evidence suggests that skeletal muscle alterations could also contribute to the onset and progression of ALS. This latter event is preceded by the decommissioning of the neuromuscular junction [63, 64], where a toxic role for skeletal muscle has been suggested [65]. Indeed, the role of skeletal muscle in activating a retrograde signalling cascade that negatively affects motor neuron health is a current topic of investigation in the pathogenesis of ALS.

In ALS, skeletal muscle degeneration is a complex scenario in which different players could be involved [66]. Ultrastructural studies performed as early as the 1960s on muscle autopsy/biopsy samples from ALS patients revealed remarkable mitochondrial abnormalities in terms of morphology, quantity, and disposition [67]. Moreover, mitochondrial abnormalities were commonly observed in the soma of the neurons in the spinal ventral horn of patients [68] and in animal models of ALS, including transgenic mice with overexpression of various ALS-associated human SOD1 mutations [69, 70]. In general, mitochondria numbers were depressed [71]. Swollen mitochondria characterized by disorganized cristae were ectopically detected in the inter-filamentous space [67, 71, 72]. Mitochondria with altered morphology also showed impaired oxidative metabolism and reduced activity of specific enzymes of the respiratory chain complex, such as NADH dehydrogenase [73] and cytochrome c oxidase (COX) [74]; all these phenomena were linked to reduced maximal respiration capacity. The relevance of altered COX activity was further confirmed by histochemical and biochemical studies on muscle biopsies from ALS patients that demonstrated a correlation with the progression of ALS [75]. Abnormalities in mitochondrial DNA were also reported [76]. The expression of genes involved in mitochondrial oxidative phosphorylation, ATP synthesis [77], and mitochondrial biogenesis were impaired [78]. The similarity of the defects in morphology and biochemical properties of mitochondria reported in muscle samples from both sporadic and familial ALS patients suggests that the mitochondria are primary players in neuromuscular degeneration despite the etiology of the pathology [66].

The underlying pathogenic mechanisms responsible for impaired mitochondrial function were investigated in studies performed in ALS rodent models [79, 80]. In skeletal muscle from SOD1^G93A^ transgenic mice, a model that expresses a mutant SOD1 carrying the G93A missense mutation and which develops an ALS-like motor neuron disease [79], the reduced mitochondrial dynamics and network were associated with increased ROS generation [81], in the absence of motor neuron axonal withdrawal, suggesting that muscle mitochondria could be directly affected by a mutation localized into the skeletal muscle [82].

Increased autophagy activity has been reported in the spinal cord of ALS patients and ALS animal models [83-85], suggesting that it could have a role in the control of mitochondrial quality [86]. Although the experimental data are still quite contradictory, a significant alteration in autophagy in SOD1^G93A^ mice was observed. The dysregulation of mitochondrial dynamics, coupled with an excess of ROS generation and reduced autophagy capacity, which occurs early and becomes severe during ALS progression, could account for the impaired clearance of altered mitochondria, that ultimately could be the promoter of subsequent mitochondrial damage, thus triggering a vicious cycle.

Some mitochondrial dysfunctions are linked to the regulation of the mitochondrial inner membrane potential (ΔΨm) or calcium (Ca^2+^) homeostasis in the depolarized mitochondria. A loss of ΔΨm, leading to a decrease of the physiological driving force necessary for adenosine triphosphate (ATP) synthesis, was observed in the region surrounding the neuromuscular junction (NMJ) in muscle fibres derived from SOD1^G93A^ mice at the onset phase of ALS [87]. This finding is consistent with data showing decreased ATP levels in skeletal muscle of different ALS animal models [88, 89]. The reduced mitochondrial Ca^2+^ uptake with a subsequent elevation of the cytosolic Ca^2+^ transient [90] could be ascribed to a collapsed ΔΨm and/or a decreased protein level [91] or efficiency of the ATPase SERCA1 [92, 93]. Elevated intracellular Ca^2+^ levels could also promote the aggregation of mutant SOD1^G93A^ protein inside the mitochondria, resulting in reduced mitochondrial dynamics [81] and increased ROS generation, thus exacerbating the muscular mitochondrial damage. A dramatic increase in mitochondrial ROS production occurs after the denervation of skeletal muscle [94]; this event could be due to a loss of the physiological Ca^2+^ uptake capacity by mitochondria, which likely represents an initial trigger for mitochondrial permeability transition pore (mPTP) prolonged opening [66].

### 3.4 Body Weight Changes in ALS Patients

Weight loss is a typical clinical feature of ALS patients. It is at least in part due to dysphagia. However, besides dysphagia, other not yet fully elucidated disease-specific mechanisms could be involved, including i) a higher waste of energy because of the muscle fasciculation; ii) increased respiratory efforts and metabolism; iii) and reduced food intake [95-98]. Weight loss is recognized as a negative prognostic factor in ALS patients, whilst increased body fat, higher body mass index (BMI), and hyperlipidemia are considered protective determinants [99-102]. Several retrospective studies have attempted to investigate the role of the above-mentioned elements in ALS onset and progression, but the data are still controversial and need to reach a better understanding. Regarding body weight, different studies show that faster weight loss is associated with accelerated ALS functional rating scale (ALS-FRS-R) decline [103-106].

Accordingly, subjects with weight loss (defined as a decrease of body weight >3 kg since the disease onset) differed significantly from no-weight loss patients in terms of “vitality”, which reflects their condition of feeling more often exhausted, tired and spiritless, independent of respiratory distress, and the stage of the disease [95]. Concerning BMI, some cohort studies have shown that BMI at baseline was inversely associated with ALS risk [100] and positively with patient survival [107]. This may arise from the fact that normal weight or underweight subjects were more sensitive to a rapid BMI decline because of hypermetabolism [107] and increased resting energy expenditure [98], which may be caused by changes in skeletal muscle metabolism, as observed in ALS mouse models [102, 108] and hypothalamic alterations [109]. An interesting report shows that BMI changes in ALS patients compared to normal subjects seem to have occurred already decades long before the motor clinical manifestations [104]. The role of lipid metabolism in ALS is still controversial and debated. It is not completely clear whether the BMI decrease is correlated to a reduced risk of hyperlipidemia. Some studies have highlighted that dyslipidemia might be a protective factor in ALS patients, as a high LDL/HDL ratio was significantly associated with a survival advantage [101, 102]. Conversely, other studies did not recognize the lipid ratio as an independent predictor of survival [107].

A recent study analyzed the plasma lipidome of ALS patients to identify metabolic changes useful to monitor the disease progression and stratify the patients [110]. ALS patients show increased TG plasma levels and LDL/HDL ratios compared to control subjects. This likely is caused by the hypermetabolic condition, characterized by de novo TG hepatic biosynthesis and delivery from adipose tissue depots to skeletal muscle. Accordingly, in ALS experimental models, increased lipid has been observed in skeletal muscle consumption prior to motorneuron death [108]. Moreover, the typology of plasma lipids also significantly correlates with the specific involvement at onset as well as bulbar, spinal or respiratory [110], suggesting a lipidome analysis as a possible discriminating tool for ALS diagnosis and progression.

### 3.5 Neuroinflammation and Astrogliosis in ALS

Neuroinflammation in ALS has been reported both in animal models and in patients in the very early stages of the disease [111] and it is characterized by activated microglia, astrogliosis, and infiltration by the immune system cells into the sites of neuronal injury. Although studies have primarily focused on clarifying the role of neuroinflammation only within the central nervous system (CNS), recent evidence suggests the involvement of complex crosstalk between the CNS and peripheral immune cells [112, 113].

Neuroinflammation in ALS consists of two phases. The initial neuroprotective phase is characterized by a anti-inflammatory compensatory response by adjacent glial and/ or peripheral immune cells; by contrast, the cytotoxic phase is linked to neurodegeneration [114].

In patients with ALS, microgliosis occurs specifically with motor neuron injury in the motor cortex, along the corticospinal tract, and in the ventral horn of the spinal cord [114].

In peripheral blood of ALS patients, alterations in T lymphocytes, monocytes, complement activation, and cytokines have all been documented [112]. Activated macrophages also have been shown to increase in number in the ventral root and sciatic nerve of ALS mice [115]; these findings suggest that inflammation may also participate in NMJ dissociation during ALS progression. It is of some significance that in the sciatic nerve, macrophage activation and the inflammatory response occur as early events, which precede the onset of symptoms or clinical signs of motor weakness; thereafter, macrophage activation and inflammation progressively increase through to the end-stage [116]. These findings are reinforced by a recent demonstration of activated inflammation and abnormal glial cell responses in the limb muscle of an ALS rat model, characterized by near-denervated NMJs [117].

Some researchers propose that peripheral activated monocytes/macrophages may also contribute to the progression of ALS by infiltrating into the CNS [112] and establishing astrogliosis [118].

In ALS, astrogliosis occurs both in the spinal cord, where it is particularly evident at the level of the anterior horns, but also in the cortex, where the distribution of reactive astrocytes is comparable with that observed in aging and ischemic brains [119].

Reactive astrocytes exposed to motor neuron degeneration and inflammation in ALS undergo complex remodeling, which results in their loss of beneficial functions and gain of neuropathogenic (neurodegenerative) properties. In ALS patients, reactive astrocytes surround both upper and lower degenerating motor neurons and play a key role in ALS associated neuropathologies, including influencing motor neuron fate as well as disease progression through secretion of soluble factors [120]. In addition, these reactive astrocytes present increased expression of (i) glial fibrillary acidic protein (GFAP), (ii) Ca^2+^ binding protein S100β, (iii) cyclooxygenase-2 (COX-2), (iv) inducible nitric oxide synthase (iNOS), and (v) neuronal NOS (nNOS) [119, 121]. These phenomena may predispose the increased release of pro-inflammatory cytokines and inflammatory mediators linked to astrocyte-mediated toxicities.

Evidence suggests that another astrocyte modification, resulting in loss of TDP-43 function, may contribute to motor neuron degeneration in some ALS models [122].

Astrocytes have been shown to down-regulate the EAAT2 transporter in ALS patients [123] and increase the release of D-serine (a co-agonist of NMDA receptors), thereby supporting the hypothesis that increased glutamatergic neurotransmission is one of the pathogenetic mechanisms of astrocyte-mediated neurotoxicity. Data arising from experiments using ALS mutant human SOD1 (hSOD1) transgenic mice indicate a correlation between significant changes in astrocyte biology and motor neuron degeneration, further supporting the concept of astrocyte involvement in ALS neuropathologies [111, 113].

### 3.6 Pathogenesis of Respiratory Failure in ALS

Respiratory failure is the most common cause of death in ALS patients, and even though no reliable method to predict its onset is available, certainly, respiratory muscles and the diaphragm are invariably affected in ALS [124]. Respiratory failure usually occurs in the late stage of ALS progression, but in a small percentage of cases (< 3%), the disease can start directly with respiratory muscle weakness and unexplained hypercapnia [125]. The respiratory complications in ALS may be initially mild and then progress towards severe dysfunction. Mild respiratory involvement interferes with quality of life, sleep disturbances, and fatigue in daily activities. In a study published in 2001 [126], it was shown that ALS patients with sleep apnea had significant deficits in cognitive function, including memory and executive function. These findings suggest that nocturnal hypoxemia could cause cognitive deficits. In addition, serious hypoventilation reduces bronchial clearance and facilitates lung infection, leading to death [127]. ALS also reduces cough effectiveness through the impairment of glottis closure and expiratory muscle function, impairing the fundamental protective reflexes for airway and bronchial clearance. Moreover, in ALS patients, there is a high risk of acid aspiration due to pharyngeal and laryngeal muscle weakness.

Exacerbated diaphragm weakness could result in elevated hypoxemia, carbon dioxide retention and irreparable respiratory failure [128, 129].

### 3.7 ALS Treatments and Therapies

Despite the growing knowledge of ALS etiology, the complexity of this disease has compromised the identification of disease-modifying drugs or effective neuroprotective agents. The current standard of care includes only two drugs, riluzole and edaravone. Other interventions aim to improve the quality of life of patients and their caregivers, but they are only symptomatic and palliative treatments [16].

#### 3.7.1 Disease-modifying Drug Treatments

##### 3.7.1.1 Riluzole

In 1995, the Food and Drug Administration (FDA) approved riluzole as the standard treatment for ALS patients. Approval was based on the results of two independent trials demonstrating prolonged survival or a two to three-month delay in the need for tracheostomy in the riluzole-treated group compared to controls [130, 131]. These benefits were mainly reported in patients with moderate functional impairment [26, 132].

Riluzole is a neuroprotective drug that inhibits the presynaptic release of glutamate, inactivates NMDA receptors, and triggers extracellular glutamate uptake [133, 134]. The drug is generally well tolerated at the dose of 50 mg twice daily, but racial, gender, and individual metabolic variations may lead to absorption variability and enhanced toxicity (Rilutek, prescribing information, 2018). The most common side effects were hepatotoxicity, asthenia, nausea, gastrointestinal upset, and decreased lung function, which in some patients can lead to interstitial lung disease (Rilutek, prescribing information, 2018) [135, 136].

##### 3.7.1.2 Edaravone

Edaravone is an antioxidant and scavenger of free radicals first approved in Japan in 2001 for the treatment of cerebral embolism and acute ischemic stroke [136-138].

Given the presence of excessive oxidative stress and the involvement of SOD1 in the pathogenesis of ALS, edaravone was initially tested in SOD1^G93A^ mice, which slowed motor decline and SOD1 protein aggregations, promoting cell survival and inhibiting apoptosis [139, 140].

Edaravone was approved by the FDA in 2017, although its exact mechanism of action is still unknown, and its clinically positive effects are still a matter of debate. In fact, it was tested in a highly selected cohort of patients characterized by an early onset and rapidly progressive disease [141, 142].

Edaravone is an expensive treatment with a complex dosage: 60 mg IV administration for 14 days followed by 14 days without drugs [136]. Nevertheless, this drug is well-tolerated, in fact, the primary adverse events are contusion, headache, and dermatitis [143].

#### 3.7.2 Treatment of Specific Symptoms

##### 3.7.2.1 Dietary Supplementation

Dysphagia and hypermetabolism are two key conditions of ALS prognosis that lead patients to dehydration, worsening of weakness and fatigue, and weight loss. Therefore, the modification of food consistency accompanied by the use of appropriate adaptive eating utensils, the pharmacological reduction of sialorrhea, and the presence of a nutritionist are essential from the onset of the disease [144]. When the patient becomes unable to eat independently, the same measures are carried out by assisted feeding until percutaneous endoscopic gastrostomy (PEG) becomes necessary [145, 146].

##### 3.7.2.2 Emotional Lability and Psychological Support

Emotional lability, also known as pseudobulbar affect (PBA), is the tendency of 20-50% of patients to laugh or cry inappropriately [147, 148]. Normally, especially in patients without cognitive impairment, emotional lability is a transient phenomenon, which resolves over several months, but it could be treated with a tricyclic antidepressant or the combination of dextromethorphan and quinidine [136].

Moreover, starting from the communication of the diagnosis, and for the entire duration of the disease, psychological support is crucial

In fact, as a consequence of the patient's awareness of their progressive, disabling, and terminal illness, it is common for the patient to experience depression and anxiety. Although supportive counselling is important, the use of tricyclic antidepressants and inhibitors of serotonin reuptake (amitriptyline, escitalopram and mirtazapine), bupropion and benzodiazepines are essential treatments [26, 147].

##### 3.7.2.3 Exercise

Muscle cramps, fatigue, and spasticity are some of the symptoms that progressively worsen in ALS patients. These symptoms, which are in part caused by the pathology and could be side effects of riluzole treatment, could be controlled by levetiracetam, baclofen, tizanidine, and botulinum toxin [147-149].

Although the role of physical activity is controversial, the involvement of a physiotherapist and a physiatrist are essential in the multidisciplinary team [45, 147].

##### 3.7.2.4 Respiratory Symptoms

Respiratory failure is the final outcome of ALS, with or without pneumonia. The atrophy of respiratory muscles is determined by combined degeneration of central and peripheral respiratory centres and by motor neuron degeneration. The appearance of respiratory problems could emerge at the beginning of the disease or by its progression, with the manifestation of dyspnoea, orthopnoea, daytime somnolence, morning headache, lack of restorative sleep, and frequent nocturnal waking [147].

Starting from physical techniques, such as breath stacking and assisted cough, and using bronchodilators and anti-cholinergic agents [150], worsening respiratory conditions leads to progressively more aggressive management.

Invasive mechanical ventilation through tracheostomy is the last intervention that could prolong survival but at the same time, contributes to a severe loss of quality of life (“locked-in” condition) [26, 147, 151].

#### 3.7.3 Cellular Therapies

Given the central role of neuroinflammation and astrogliosis in ALS, ongoing or completed clinical trials are focused on microglia/macrophage molecules and on new therapies aimed at modulating astrocyte biology [111, 152].

For instance, it has been observed that the progression of the disease could be slowed down by transplanting astrocyte precursors into the cervical spinal cord of SOD1^G93A^ mice [111, 153]. Moreover, cell therapy is arousing significant interest among clinicians and researchers due to extensive progress in preclinical and early phase clinical trials, since stem cells may intervene simultaneously on several mechanisms. In fact, stem cell therapy could sustain neuroprotection both by replacing damaged cells and by providing trophic support to the injured environment and also by actively participating in the regulation of neuroinflammatory processes [154, 155].

## 4 GHRELIN AND GROWTH HORMONE SECRETAGOGUES AS POTENTIAL TREATMENT FOR ALS

Patients with ALS could present metabolic abnormalities, such as weight loss, a faster rate of reduction in BMI, and hypermetabolism, which contribute to accelerating disease progression, but the correlation between the levels of eating peptides and the clinical prognosis is still largely unknown [156].

Only a few studies have assessed the plasma levels of metabolic proteins and adipokines in patients with ALS. In particular, Ngo and colleagues have demonstrated that plasma levels of active ghrelin were significantly lower in ALS patients than in their controls, and this was most pronounced in female ALS patients. Ngo and colleagues have suggested that low ghrelin levels could contribute to a decrease in total food intake and consequently to reduced survival in ALS [157].

More recently, Nagaoka and colleagues have demonstrated for the first time a shorter survival of ALS patients with a low-plasma level of ghrelin (<15 fmol/mL) compared with those with higher ghrelin levels (≥15 fmol/mL), which also correlates with a reduced number of ghrelin-secreting cells in the stomach. However, in the difference from the data obtained by Ngo, Nagaoka observed a significantly lower level of ghrelin in the ALS-male group than in the ALS-female group, but each single gender group of ALS patients had ghrelin levels that were no statistically different from the control group [10].

Therefore, these studies demonstrate that ghrelin levels are associated with increased survival of ALS patients, but further studies are required to fully elucidate the neuroendocrine/metabolic pathophysiology of ALS and to examine the impact of various nutritional interventions on ALS patients.

### 4.1 Modulation of Neuronal Pathology and Neuroinflammation

Evidence suggests that ghrelin may exert neuroprotective effects in the CNS [158]. Recently, it has been shown that ghrelin and hexarelin are endowed with anti-oxidant, anti-inflammatory, and anti-apoptotic effects [159] in different *in vivo* and *in vitro* disease models, including stroke [160, 161], Parkinson’s disease [162, 163], Alzheimer’s disease [164], multiple sclerosis [165], and ALS [166-168]. The expression of GHS-R1a in the spinal cord suggests that ghrelin could have direct effects on spinal cord function. In the spinal cord, the IGF-1 system may be involved in the neuroprotective effect of ghrelin, which prevents apoptotic death of motoneurons [169]. Ghrelin and hexarelin modulate the phosphatidylinositol-3-kinase (PI3K)/Akt and extracellular signal-regulated kinase 1/2 (ERK1/2) pathways, both of which are involved in cell survival [50, 159, 170]. Collectively, these findings suggest that ghrelin and synthetic GHS have the potential to become novel options for the treatment of ALS [166].

In neurodegenerative diseases, neuroinflammation is frequently associated with reactive microglial and astroglial cells, infiltrating lymphocytes and macrophages, and activation of the complement system [171, 172].

Some evidence suggests that in ALS, the immune system may dynamically balance between neuroprotection and neurotoxicity. Specifically, during periods of slow disease progression, the immune system exerts a protective effect by secreting anti-inflammatory factors that rescue and repair damaged tissue; by contrast, during accelerated disease progression, the immune system exerts a strong proinflammatory and neurotoxic effect [114].

In many neurodegenerative diseases, the Nod-like receptor protein 3 (NLRP3) inflammasome participates in an innate initiation of the immune response [173-175]. The activation of the NLRP3 inflammasome generates pyroptosis, a recently discovered form of inflammatory cell death [176]. Downstream target proteins in pyroptosis are interleukin (IL)-1β and IL-18, which are activated after the release of functional caspase-1. As a result, cells swell until their membranes burst, releasing a large number of inflammatory cytokines, which leads to a dangerous inflammatory cascade [176].

The expression, activation, and co-localization of the NLRP3 inflammasome have been investigated in the spinal cord of animal models of ALS, as well as in post-mortem tissue of ALS patients; significantly, both NLRP3 and its molecular components are detectable in pre-symptomatic animals, in spinal cord astrocytes and in human tissue (compared to control patients) [177]. These findings suggest that astroglial NLRP3 inflammasome complexes contribute to disease progression and may therefore be a potential target to arrest microglial neuroinflammation and ALS disease progression [178].

**Fig. (1):**
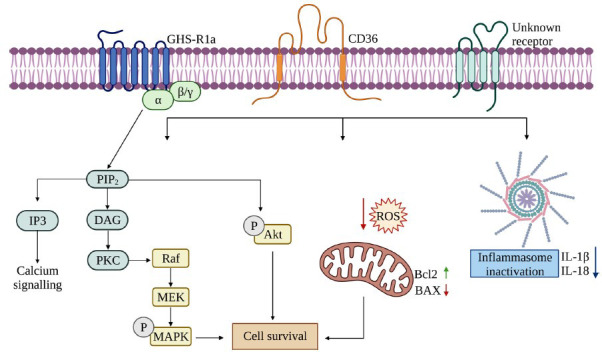
A schematic drawing of the major neuroprotective signaling pathways regulated by ghrelin and GHS. The canonical pathway of ghrelin and GHS activities involves their binding to the GHS-R1a receptor. GHS-R1a activation results in the hydroxylation of phosphatidylinositol 4,5-bisphosphate (PIP2) to generate the secondary messengers inositol 1,4,5-triphosphate (IP3) and diacylglycerol (DAG), which lead to mobilization of intracellular calcium (Ca^2+^) stores and to protein kinase C (PKC) activation. PKC mediates different cellular responses, including the stimulation of mitogen-activated protein kinases (MAPKs), and the protein inositol 3 kinase (PI3K)/Akt pathway. The alternative pathways of ghrelin and GHS activities include the binding and activation of CD36 or other unknown receptors, which stimulates the activation of MAPKs and Akt, inhibits the generation of reactive oxygen species (ROS) and reduce the activation of BAX, a pro-apoptotic molecule, suppressing apoptosis and improving cell survival. Ghrelin and GHS also attenuate neuroinflammation involving the NLRP3 inflammasome signaling pathway and pyroptosis. Created in Biorender.com.

Interestingly, astrocytes express the GHS-R1a-receptor and are responsive to ghrelin [179]. Ghrelin acts as a survival and anti-inflammatory factor through stimulation of ERK1/2, PI3K/Akt, and the anti-apoptotic protein B-cell lymphoma 2 (Bcl-2), as well as by inhibiting caspase-3 [179]. In addition, ghrelin markedly attenuates inflammation and inhibits inflammatory factors and microglial activation by suppressing the NLRP3 inflammasome signalling pathway and pyroptosis [165] (Fig. **1**).

### 4.2 Role in Skeletal Muscle

Clinical hallmarks of ALS include progressive muscle wasting, characterized by muscle atrophy, speech and swallowing difficulties, fasciculation, altered reflexes, spasticity and ultimately paralysis.

Several studies have proposed that muscles may deliver protective molecules such as neurotrophins to motor neurons in a retrograde fashion and have attempted to mitigate muscle pathology to counteract motor neuron degeneration. Different therapeutic strategies focused on various targets have been proposed [180]. Some studies have documented depressed IGF-1 levels in muscle from ALS patients [181]; by comparison, elevated levels of muscle IGF-1 in mutant SOD1^G93A^ mice were linked to improved muscle function and increased motor neuron survival [182-185]. Promising results from preclinical studies with IGF-1 may open new perspectives in ALS management. IGF-1 is a key anabolic growth factor for regulating muscle hypertrophy, sharing some growth and development stimulatory properties with ghrelin and GHS.

The myotrophic effects of ghrelin and GHS have been described in different pathological conditions. Muscle wasting is a co-morbidity associated with many disorders that severely increase morbidity and affect patient prognosis and quality of life. The loss of skeletal muscle, often accompanied by a reduction in appetite, increased catabolism, and loss in body weight, is a key adverse factor in cancer cachexia. Muscle wasting is a predisposing factor in the clinical decline of patients, is directly associated with increased mortality, and represents a negative predictor of treatment outcome [186]. Ghrelin, hexarelin, and JMV2894 in preclinical models of cisplatin-based chemotherapy improved not only anorexia and weight loss but also strength and mass muscle, as well as increased survival [167, 187, 188]. Ghrelin and GHS reduced muscle wasting through multiple mechanisms of action [167]. The substrates for cachexia, although not fully elucidated, are multifactorial and dependent on different mechanisms, including (i) activation of the ubiquitin (Ub)-proteasome and autophagy systems, (ii) reduced protein synthesis, (iii) activation of p38/C/EBP-β and Mstn, (iv) an increase in inflammatory cytokines, and (v) the downregulation of Akt and myogenin/MyoD. Ghrelin, hexarelin, and JMV2894 prevent muscle atrophy by mitigating inflammation and p38/C/EBP-β/Mstn activation, thereby increasing Akt phosphorylation as well as myogenin and MyoD expression [189]. Additional effects include downregulation of muscle RING-finger protein-1 (Murf-1) E3 transcript expression, which is a key component of the proteasome system [190]. Ghrelin, hexarelin, and JMV2894 increase the transcriptional coactivator peroxisome proliferator-activated receptor γ coactivator-1α (PGC-1α), which is a marker of muscle oxidative phenotype [167, 188, 191]. Ghrelin, hexarelin, and JMV2894 have also been associated with attenuation of muscle damage as reflected in increased cross-sectional area of myofibers, which contributed to the measured improvement of forelimb force [192]. Ghrelin has also been shown to prevent muscle atrophy induced by dexamethasone and angiotensin II [189, 193, 194].

### 4.3 Modulation of Muscle Mitochondrial Homeostasis

There is very little information on the roles that ghrelin and GHS play in muscle mitochondrial homeostasis; investigations have focused largely on mitochondrial oxidative capacity/stress, dynamics, and calcium regulation.

Short-term treatment with ghrelin increased mitochondrial enzyme activities and UCP-2 expression and thereby improved oxidative capacity in C2C12 myoblasts *in vitro* [195], as well as in healthy and pathological rat skeletal muscle *in vivo* [196-198]. Ghrelin treatment did not modify obesity-induced skeletal muscle redox state and did not increase muscle glutathione peroxidase (GPx) or glutathione oxidation status [199]. In comparison, desacyl ghrelin reduced mitochondrial ROS generation in neonatal ventricular myocytes [200] in both healthy as well as pathological rodent models [201-203].

Our understanding of GHS effects on muscle Ca^2+^ homeostasis and mitochondrial dynamics has been derived largely from studies performed in a rat cachexia model induced by cisplatin (CDDP). CDDP increases intracellular resting Ca^2+^ levels in isolated extensor digitorum longus (EDL) muscles. As a consequence, cellular responsiveness to stimulation by depolarizing solutions, as well as caffeine, is reduced. In this regard, caffeine is notable as it is a modulator of the RyR channels and thereby affects store-operated Ca^2+^ entry (SOCE), which is essential for proper muscle function [7]. CDDP also affects the expression of genes involved in Ca^2+^ homeostasis in skeletal muscle, such as the RyR-type 1(RyR1) Ca^2+^-channel, the stromal interaction molecule (Stim1) and its main target, the Ca^2+^ release-activated Ca^2+^ modulator 1 (Orai1) [7]. In this context, it is significant that some GHS, such as JMV2894 and hexarelin, effectively inhibit CDDP disruption of Ca^2+^ homeostasis and consequently reduce resting intracellular Ca^2+^ levels; this normalization of Ca^2+^ homeostasis restored expression of RyR1, Stim1, and Orai1. Ultimately, SOCE activity was restored, which allowed myofibers to recover their responsiveness to caffeine [191]. JMV2894 treatment also prevented CDDP-induced mitochondrial damage and preserved muscle mitochondrial function, morphology, distribution, and turnover [7].

### 4.4 Role in Respiratory Failure

Evidence for the anti-inflammatory actions of ghrelin precipitated investigations into possible roles for ghrelin in the respiratory system. Indeed, ghrelin inhibited the expression of proinflammatory cytokines released by monocytes and endothelial cells in an experimental model of endotoxic shock [204]. In particular, Li *et al.* [204] demonstrated that ghrelin reduced chemotactic cytokine production and mononuclear adhesion in human vascular endothelial cells *in vitro*. In an endotoxic model, ghrelin reduced levels of interleukin (IL)-8 and tumour necrosis factor (TNF)-α through activation of GHS-R1a. In addition, ghrelin administration modulated sepsis-induced lung injury (through inhibition of nuclear factor (NF)-κB), reduced lung water content, and decreased pro-inflammatory cytokines, which resulted in improved survival [205]. Similar beneficial effects were observed in other experimental studies on lipopolysaccharide (LPS)-induced lung injury, which demonstrated that the anti-inflammatory action of ghrelin depends on increased nitric oxide production [206] and the anti-apoptotic effects of alveolar macrophages [207]. Ghrelin also has anti-fibrotic effects on the injured lung. In an experimental model of bleomycin-induced injury, ghrelin administration maintained the alveolar epithelial cell layer; this effect was followed by suppression of fibroblast proliferation and matrix deposition, as well as reduction of IL-1β and IGF-1levels [208]. Ghrelin treatment is also effective in reducing lung injury in an elastase-induced emphysema model in mice. This protective effect supported improved cardiovascular function and reversed the loss of body weight [209]. Moreover, total increased collagen deposition in the lung parenchyma (typical of mild to moderate and severe chronic obstructive pulmonary disease (COPD) patients; [210]) was significantly reduced in ghrelin-treated animals. These data were confirmed in our recent study on the treatment with hexarelin in an experimental model of unilateral acid aspiration: we demonstrated that hexarelin administration can reduce the early inflammatory response in the lung induced by acid instillation with the consequent decrease of lung fibrosis [211]. The lung-protective effects of ghrelin appear to be mediated in part by the endoplasmic reticulum, as revealed in a study using an oleic-treated acid rat model, a well-characterized and clinically relevant animal model of acute lung injury [212]. By comparison, ghrelin protective effects on the lung were associated with reductions in levels of transforming growth factor (TGF)-β1, matrix metalloproteinase (MMP)‐2 and accumulations of extracellular adenosine in pulmonary tissue in a lung-contusion model [213]. In instances of ventilator-induced lung injury, lung-protective effects of ghrelin were associated with reductions of Toll-like receptor (TLR)-4 and NF-kB expression [214].

In clinical studies, ghrelin suppressed neutrophil recruitment into the airways and improved symptoms and the respiratory strength of cachectic COPD patients [215, 216].

## CONCLUSION

Existing treatments for ALS yield only minimal benefits to patients and their survival, therefore, there is a pressing need for more effective treatment strategies. Ghrelin and GHS have been shown to be generally safe for use in humans. In light of the known substrates for ALS, evidence from studies on the physiological and molecular basis of ghrelin/GHS bioactivities suggests a potential role for ghrelin and GHS in the treatment of ALS. Indeed, there are ongoing trials on the use of ghrelin and GHS for the treatment of various neurodegenerative diseases [2].

Starting from 2001, four different patents have proposed the use of ghrelin or analogues as preventive/therapeutic agents for neurodegenerative diseases (WO2001047558A1, WO201406534A1, WO2015017123A1, WO2017075535A1). Among them, WO2014065341A1 proposed ghrelin or its mimetics as therapeutic agents for the treatment of ALS, in combination with current therapies, in patients that do not exhibit severe dysphagia and are insufficiently responsive to existing therapeutic agents for ALS. The results have demonstrated that tested treatments had motor neuron protective actions and suppressed muscular weakness and strength reduction, suggesting that a pharmaceutical composition comprising a GHS receptor agonist can be a potential therapeutic agent for ALS.

Finally, from April 2021, the Japanese Health Authorities has approved Adlumiz (anamorelin), an orally active, high-affinity, selective ghrelin receptor agonist, for the treatment of anorexia, cachexia or unintended weight loss in adult patients with a non-small cell lung cancer (NSCLC). Adlumiz has shown efficacy in increasing body weight, muscle mass, and appetite in cancer cachexia patients; the risks identified are justified by the benefits. Previously, in 2017, the European Medicine Agency (EMA) refused the granting of the marketing authorization of anamorelin because its safety and efficacy were not sufficiently demonstrated; actually, the development of this drug is ongoing internationally, through two phase III SCALA studies (ANAM-17-20: https://clinicaltrials.gov/ct2/show/NCT03743051; ANAM-17-21: https://clinicaltrials.gov/ct2/show/NCT03743064).

In the context of potentially novel and more effective ALS therapies, comprehensive investigations of ghrelin and GHS modulation of apoptosis and inflammation, mitochondrial dysfunction and Ca^2+^ homeostasis are timely.

## LIST OF ABBREVIATIONS

ALS: Amyotrophic Lateral Sclerosis
AMPA: α-Amino-3-hydroxyl-5-methyl-4-isoxazole-Propionate
ATP: Adenosine Triphosphate
BBB: Blood-brain-barrier
BCL-2: B-cell Lymphoma 2
BMAA: β-methylamino-L-alanine
Ca^2+^: Calcium
CDDP: Cachexia Model Induced by Cisplatin
CNS: Central Nervous System
COPD: Chronic Obstructive Pulmonary Disease
COX: Cytochrome c Oxidase
COX-2: Cyclooxygenase-2
CSF: Cerebrospinal Fluid
EAATs: Excitatory Amino Acid Transporters
EDL: Extensor Digitorum Longus
ERK1/2: Extracellular Signal-regulated Kinase 1/2
ETM4: Hereditary Essential Tremor-4
fALS: Familial-type Amyotrophic Lateral Sclerosis
FDA: Food and Drug Administration
FTLD: Frontotemporal Lobar Degeneration
FUS/TLS: Fused in Sarcoma/Translocated in Sarcoma
FVC: Forced Vital Capacity
GFAP: Glial Fibrillary Acidic Protein
GH: Growth Hormone
GHRP-6: Growth Hormone-releasing Peptide-6
GHS: Growth Hormone Secretagogues
GHS-R1a: GH-secretagogue Receptor
GluR2: Subunit 2 of AMPA Receptors
GPx: Glutathione Peroxidase
GWAS: Genome-wide Association Studies
hSOD1: Human SOD1
IGF-1: Insulin-like Growth Factor 1
IL: Interleukin
iNOS: Inducible Nitric Oxide Synthase
LPS: Lipopolysaccharide
MMP: Matrix Metalloproteinase
MND: Motor Neuron Disease
mPTP: Mitochondrial Permeability Transition Pore
mtDNA: Mitochondrial DNA
Murf-1: Muscle RING-finger Protein-1
NF-κB: Nuclear Factor-κB
NGS: Next-generation Sequencing
NIV: Non-invasive Ventilation
NLRP3: Nod-like Receptor Protein 3
NMDA: N-methyl-D-aspartate
NMJ: Neuromuscular Junction
nNOS: Neuronal NOS
NO: Nitric Oxide
Orai1: Ca^2+^ Release-activated Ca^2+^ Modulator 1
OS: Oxidative Stress
PBA: Pseudobulbar Affect
PEG: Percutaneous Endoscopic Gastrostomy
PGC-1α: Peroxisome Proliferator-activated Receptor γ Coactivator-1α
PI3K: Phosphatidylinositol-3-kinase
RNS: Reactive Nitrogen Species
ROS: Reactive Oxygen Species
RyR1: RyR-Type 1
sALS: Sporadic form Amyotrophic Lateral Sclerosis
SNIFF: Sniff Nasal Inspiratory Pressure
SOCE: Store-operated Ca^2+^ Entry
SOD1: Superoxide Dismutase 1
Stim1: Stromal Interaction Molecule
TARDBP: TAR DNA Binding Protein
TGF: Transforming Growth Factor
THA: Threohydroxyaspartate
TLR: Toll-like Receptor
TNF: Tumour Necrosis Factor
Ub: Ubiquitin
VC: Vital Capacity
